# Perceptual control models of pursuit manual tracking demonstrate individual specificity and parameter consistency

**DOI:** 10.3758/s13414-017-1398-2

**Published:** 2017-08-25

**Authors:** Maximilian G. Parker, Sarah F. Tyson, Andrew P. Weightman, Bruce Abbott, Richard Emsley, Warren Mansell

**Affiliations:** 1 Division of Psychology and Mental Health, School of Psychological Sciences, University of Manchester, 2nd Floor Zochonis Building, Brunswick Street, Manchester, M13 9PL UK; 2 Division of Nursing, Midwifery and Social Work, University of Manchester, Manchester, UK; 3 School of Mechanical, Aerospace and Civil Engineering, University of Manchester, Manchester, UK; 4 Psychology Department, Indiana University – Purdue University Fort Wayne, Fort Wayne, IN USA; 5 Centre for Biostatistics, School of Health Sciences, University of Manchester, Manchester Academic Health Science Centre, Manchester, UK

**Keywords:** Perceptual learning, Motor control, Math modeling

## Abstract

Computational models that simulate individuals’ movements in pursuit-tracking tasks have been used to elucidate mechanisms of human motor control. Whilst there is evidence that individuals demonstrate idiosyncratic control-tracking strategies, it remains unclear whether models can be sensitive to these idiosyncrasies. Perceptual control theory (PCT) provides a unique model architecture with an internally set reference value parameter, and can be optimized to fit an individual’s tracking behavior. The current study investigated whether PCT models could show temporal stability and individual specificity over time. Twenty adults completed three blocks of 15 1-min, pursuit-tracking trials. Two blocks (training and post-training) were completed in one session and the third was completed after 1 week (follow-up). The target moved in a one-dimensional, pseudorandom pattern. PCT models were optimized to the training data using a least-mean-squares algorithm, and validated with data from post-training and follow-up. We found significant inter-individual variability (partial η^2^: .464–.697) and intra-individual consistency (Cronbach’s *α*: .880–.976) in parameter estimates. Polynomial regression revealed that all model parameters, including the reference value parameter, contribute to simulation accuracy. Participants’ tracking performances were significantly more accurately simulated by models developed from their own tracking data than by models developed from other participants’ data. We conclude that PCT models can be optimized to simulate the performance of an individual and that the test-retest reliability of individual models is a necessary criterion for evaluating computational models of human performance.

The ability to control visual and proprioceptive variables underpins all human manual skills. Tracking tasks, in which an end-effector (joystick or handle) is used to keep a cursor aligned with a target that changes position over time (Poulton, 1952a, b; Poulton, 1974), have thus featured prominently in research studies of motor control and human-computer interaction. System identification approaches, applied to tracking behavior, have led to the development of general computational models of the human operator (Levison, Baron, & Kleinman, 1969; McRuer & Jex, 1967). However, it has been established that humans display idiosyncratic invariants in some movement parameters (Morasso, 1981). These characteristic individual “traits” should be evident between individuals’ manual tracking behavior and show temporal stability within individuals. Below we review the evidence for such idiosyncrasies in individual tracking performance, and outline a model derived from the perceptual control theory (PCT; Powers, 1973) that is capable of capturing these idiosyncrasies. The current study explores the potential for this computational model to individually characterize 20 individuals’ control strategies and differentially simulate their performance.

Time-series and frequency analysis of individual performance in pursuit tracking indicates that manual tracking performance is dependent on a number of factors. In the first instance, tracking strategies are partly determined by task constraints, such as the frequency of the target signal (Neilson, Neilson, & O’Dwyer, 1993) and the motion pattern of the target, for instance whether targets move in sinusoidal or pseudorandom patterns (Notterman & Tufano, 1980; P Viviani & Mounoud, 1990). Individuals also demonstrate large individual differences in tracking strategies and performance due to user-related factors, including the volume of task practice (Notterman & Tufano, 1980), previous joystick experience (Joseph & Willingham, 2000), and age (Jagacinski, Liao, & Fayyad, 1995; Liao, Jagacinski, & Greenberg, 1997). Differences are even more evident in the tracking behavior of individuals with motor deficits, such as the characteristic impairments of people with Parkinson’s disease. Individuals with Parkinson’s disease tend to undershoot the target peaks and demonstrate increased pursuit latencies relative to control participants (Aiman Abdel-Malek, Markham, Marmarelis, & Marmarelis, 1988; Flowers, 1978). The construction of dynamic models of pursuit-tracking performance in healthy and atypical populations has helped to elucidate the nature of individual differences in tracking.

In healthy populations, dynamic models optimized to the data of individual participants demonstrate that idiosyncrasies in tracking performance can be reflected in estimated model gains and time constants (delays) (Abdel-Malek & Marmarelis, 1988; Viviani, Campadelli, & Mounoud, 1987; Viviani & Mounoud, 1990). Computational models of pursuit performance in people with Parkinson’s disease have shown that patterns of parameter estimates reflect their specific impairments in motor planning and execution. The characteristic target undershoot is quantified in the model by overdamped output relative to control participants (Aiman Abdel-Malek et al., 1988; Au, Lei, Oishi, & McKeown, 2010). Timing issues are evident in delays and velocity control gains (Viviani, Burkhard, Chiuvé, Dell’Acqua, & Vindras, 2009). Analysis of these parameters (gains, delays and damping constants, optimized to individual performance) enable discrimination between samples of people with Parkinson’s in receipt of medication, those who are non-medicated, and controls, despite the absence of a difference in overall task accuracy between the groups (Au et al., 2010; Oishi, Ashoori, & McKeown, 2010). Whilst many studies found that models accurately simulated the tracking behavior of individuals in model validation tests in both typical samples (Abdel-Malek & Marmarelis, 1988; Aiman Abdel-Malek & Marmarelis, 1990; Marken, 1991; Powers, 1978; Viviani et al., 1987; Viviani & Mounoud, 1990) and Parkinson’s disease samples (Aiman Abdel-Malek et al., 1988; Au et al., 2010; Oishi et al., 2010; Oishi, Talebifard, & McKeown, 2011; Viviani et al., 2009), there is a paucity of research studies that validate models with data collected at a later time point. This is problematic because the accuracy, and therefore usefulness, of a model must be dependent on the individual’s control strategy remaining stable over time in a well-practiced individual. Whilst this has not been specifically modelled in tracking studies, there is some indication from studies of motor performance that control strategies might show temporal stability.

It has been established that movement parameters exist that are invariant over repeated movement performances within participants, despite overall variability in produced movements and individual differences between participants. These include velocity profiles and hand tangential velocity in reaching movements (Morasso, 1981), and movement trajectories in pointing and joint angle-velocity ratios in pointing (Soechting & Lacquaniti, 1981). However, there have been few studies testing whether this is the case in tracking experiments over repeated occasions. In one study, participants tracked a sinusoidal signal at a single frequency over 10 days. The variability in their pursuit velocity profiles reduced as the variability in their error decreased, assessed by the correlation coefficient between trials each day (Franks, Wilberg, & Fishburne, 1982), indicating that participants learned a particular control strategy. Another study showed that participants produced, and could be differentiated by, individual characteristic direction-velocity distribution “ensembles” in the tracking of two-dimensional sinusoidal targets, which persisted over a range of target frequencies (Miyake, Loslever, & Hancock, 2001). While these studies suggest that intra-individual consistencies in tracking strategies may exist, we found only two studies that explicitly optimized models to participants’ behavior at one time point, and validated the model with data collected at a second time point (Bourbon, 1996; Bourbon, Copeland, Dyer, Harman, & Mosley, 1990). These studies found strong correlations (r = .98) between the model-simulated tracking movements and the participants own pursuit movements. These studies had small sample sizes: five participants over 1 year (Bourbon et al., 1990) and a single case (the author) over 5 years (Bourbon, 1996). Whilst models accurately simulated the participant from which they were developed over this time period, the authors did not measure intra-individual consistency or individual differences in parameter estimates over the repeated testing sessions. The aforementioned studies used a computational architecture derived from perceptual control theory (Powers, 1973), which purports to have the potential to differentially simulate individual performance in healthy participants (Bourbon, 1996).

Perceptual control theory (Powers, 1973) is derived from conceptual principles, and therefore the functions of model parameters (on which individuals should differ) are pre-specified, and relate to specific aspects of the individual’s control strategy. This is in contrast to other models identified via system identification. The tracking model comprises the gains, delays, and damping parameters common to other theories, in addition to another, unique parameter – the reference value. This parameter represents the goal specification for the control system, and in PCT is set within the controlling system rather than from outside it (Mansell & Marken, 2015). Hence PCT provides a model of a purposeful system (Powers, 1978). Whilst other theories would assume this goal specification would be zero within the constraints of the tracking task (as participants are instructed to keep the cursor and target aligned), this is not the case when models including a reference value parameter are optimized to individual performance (Mansell & Marken, 2015). In fact, estimated reference parameters frequently hold a non-zero value (Powers, 1978, 1989). The addition of this control parameter may improve the simulation accuracy of models to individuals’ validation data and allow discrimination between individuals as their specific goal specification must be a core feature of their control strategy. Whilst PCT models have been frequently demonstrated to simulate individual performance to a high degree of accuracy in well-practiced participants (Bourbon, 1996; Bourbon et al., 1990; Powers, 1978, 1989), the nature of the relationship between PCT parameters and performance remains unknown. Moreover, whilst the findings of Bourbon et al. (1996, 1990) suggest that well-practiced individuals might demonstrate idiosyncratic patterns of control parameters that remain consistent over time (so long as task demands and constraints remain fairly stable), this has not been directly tested to date. It remains to be demonstrated whether PCT can be used to differentially simulate individual performance.

The current study aimed to examine the estimated control parameters of a PCT model optimized to individual’s performance over 1 week, and elucidate the relationship between individual parameters and model simulation accuracy. We trained a PCT model on each participant’s pursuit movements during a tracking task, and examined several factors: the reliability of estimated parameter values for each participant, individual differences between participants’ models over 1 week, and the nature of the relationship between estimated control parameter values and model accuracy. To determine whether models were individual-specific, we additionally tested whether these models could make idiographic predictions of participants’ own pursuit movements after 1 week (validation), and whether these “self” simulations were superior in accuracy to the predictions of a general aggregate “other” model (that had not been optimized to the participant’s data). We hypothesized that: (1) parameter estimates of an individual’s computational model will remain stable over time (1 week); (2) there will be differences in parameter estimates between individuals; (3) estimated parameters are suspected to hold a quadratic relationship with model simulation error, as the participants are presumed to converge on optimal parameters in the task. The reference value parameter will increase the variance explained by the regression model when added to the model consisting of the other parameters; (4) the models generated from an individual’s parameter estimates during training will accurately simulate an individual’s tracking movements even after 1 week has elapsed; and (5) a participant’s tracking data will be more accurately simulated by the participant’s own model than by other participants’ models. We expect that the difference will be small but consistent given that participants are hypothesized to converge on an optimal control strategy for this task.

## Method

### Design

**Fig. 1:**
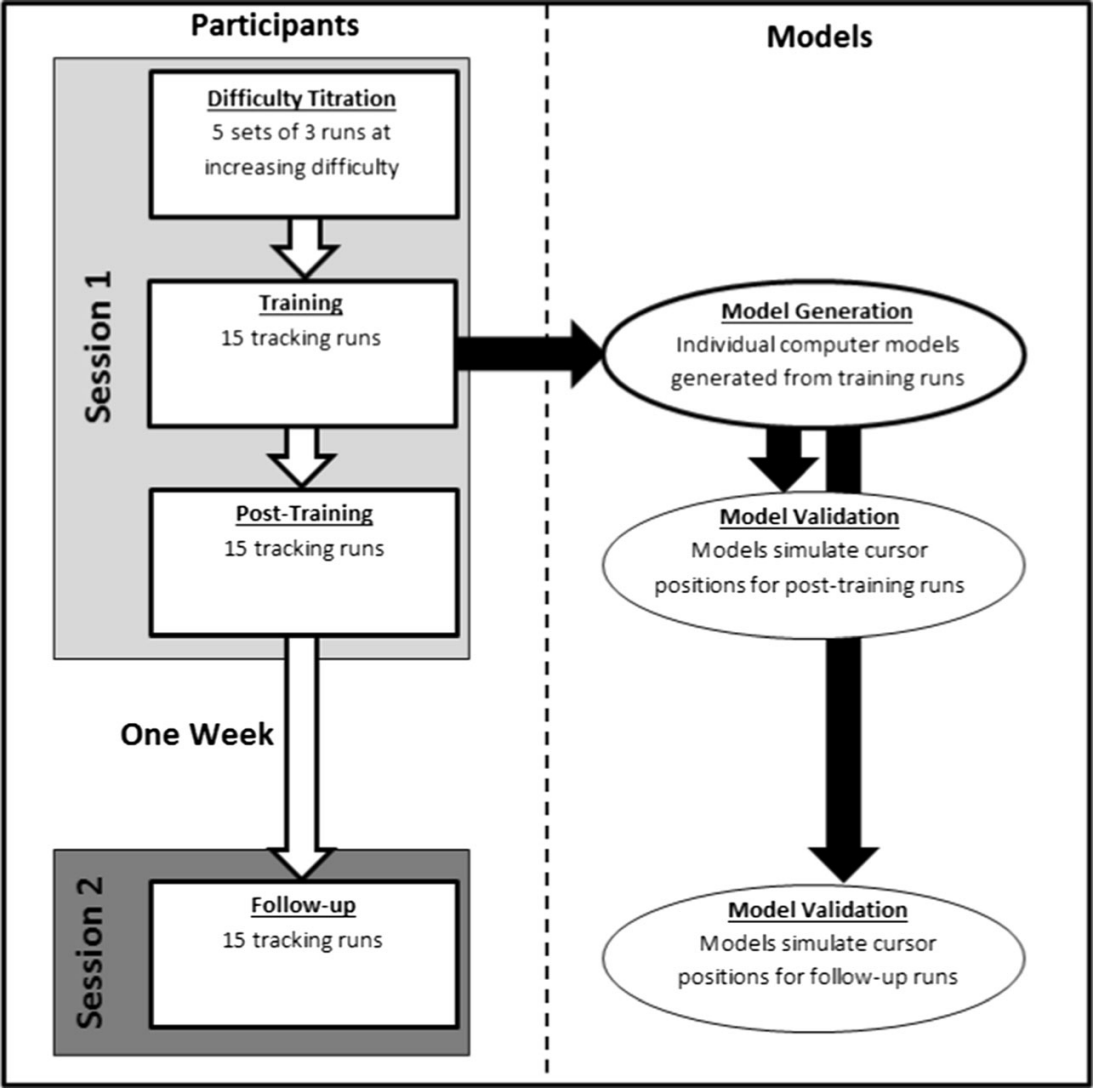
Flow diagram of the experiment design

**Fig. 2:**
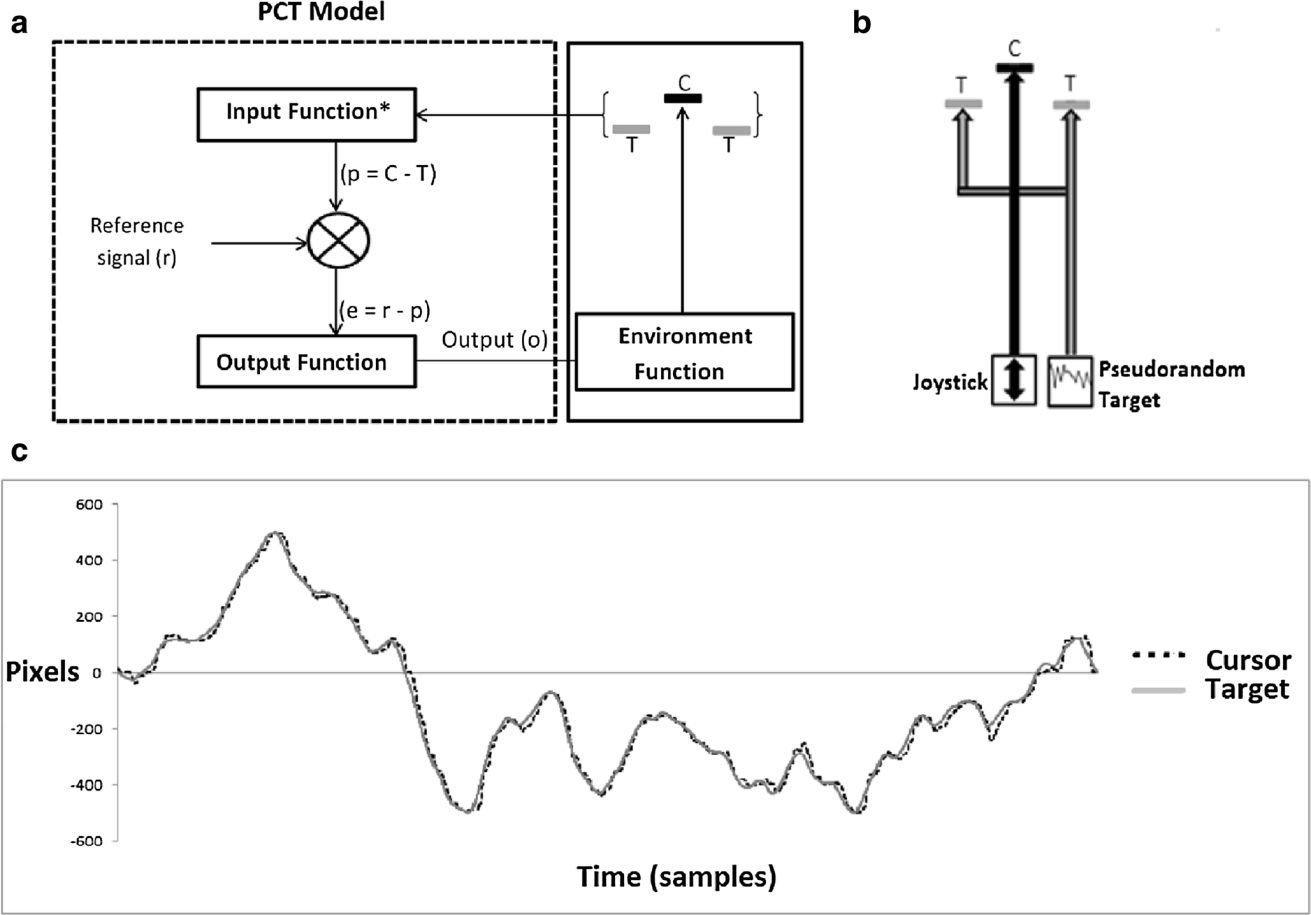
**a** The experimental setup with the computer model and screen. The computer model takes feedback from the cursor-target positional error as an input and compares this distance to the desired reference distance (*r*) between target (T) and cursor (C). **b** The experimental setup from the viewpoint of the participant. The joystick position is altered to move the cursor (C) in the vertical dimension and the target marks (T) move according to a pseudorandom pattern. **c** The results of a typical 1-min run completed by a participant. *Target* (*T*): grey line, *cursor* (*C*): black dotted line

The experiment required 20 participants to complete “runs” of a pursuit-tracking task (Fig. 2, panel b). For each “run” the participant continuously tracked a target moving in a pseudorandom pattern for 1 min. Target and cursor positions were recorded every 16.7 ms. Participants completed three blocks of pursuit-tracking runs over two sessions, separated by 1 week (Fig. 1). The first session consisted of a difficulty titration procedure (explained in full in the procedure section below), followed by the first block of 15 “training runs” (from which the model was derived), and the second block of 15 “post-training runs” (which were the benchmark for model validation). In the second session, which took place at least 1 week after the first, participants completed the third block of 15 “follow-up runs” (second validation).

Each participant’s training runs were used to generate an individual model. Each model simulated that participant’s cursor movements during the post-training and follow-up runs (Fig. 1). The participants’ tracking accuracy was assessed by root mean square error (RMSE) between the target and cursor movements over the 1-min run, expressed as a percentage of the total target excursion range (track RMSE). A second RMSE value quantified the accuracy of the fit of the model-simulated cursor movements to the participant’s actual cursor movements for each run; this was also expressed as a percentage of total target excursion range (model RMSE).

### Participants

Twenty healthy volunteers were recruited through the University of Manchester volunteer database. Participants were excluded if they had impaired, uncorrected vision or any diagnosis of a neurological problem of motor control. Participants were financially reimbursed or awarded course credits for their participation. Ethical approval was granted by the University Research Ethics Committee (UREC) at the University of Manchester (UREC Reference: 15247).

We could identify only one study with a comparable analysis to ours. They confirmed the individual differences in parameter estimates of a model in a sample of ten participants (Viviani et al., 1987). This article did not provide sufficient methodological detail for a power analysis. More recent studies of idiosyncrasies in pursuit tracking used 12 participants (Miyake et al., 2001) or groups of 20 or fewer participants (Oishi et al., 2010; Viviani et al., 2009), but did not conduct a similar analysis. As our primary aim in this article was to determine whether parameter estimates and simulation accuracy were temporally stable within individuals, it was crucial that we collect enough data from each participant. Therefore we selected a sample size of 20 participants based on previous studies of this kind, and collected tracking data from 45 runs for each participant over two sessions, although participants completed 62 runs in total.

### Apparatus

#### TrackAnalyze

The pursuit-tracking task used was the TrackAnalyze program, part of the Living Control Systems III: The Fact of Control suite (Powers, 2008). In the task, the participant uses a Microsoft Sidewinder Force Feedback 2 joystick (*J*) to keep a cursor (*C*) aligned with a moving target (*T*) in one dimension (Fig. 2b). The cursor is a green horizontal bar (black in figure) and the target marks are two red horizontal bars (grey in figure). The participant was asked to keep the green cursor positioned between the red bars. Both the target and cursor could move only in the vertical dimension. The joystick positions were sampled and scaled such that joystick position and cursor position had a directly proportional relationship (*C* proportional to *J*). A computer algorithm used a pseudo-random number generator and smoothing routine to produce the pseudorandom target time series. The algorithm generates values in the time series by multiplying a random number (rectangular distribution with mean 0 and range ±0.5), by 20,000 yielding a number between ±10,000. Each number is divided by one of five smoothing factors (64, 32, 16, 8, and 4, respectively), and added to the previous value. Thus each successive value is a weighted sum of all previous values. The resultant time series is smoothed a further two times using the same smoothing factor. Finally, target time series were rescaled to the excursion permitted for the target in screen pixels. The five smoothing factors determined the rate of change of the target time-series; targets with a higher rate of change were more difficult to track, and therefore as smoothing factor value decreased (64, 32, 16, 8, 4), the assigned difficulty level of a run increased (1, 2, 3, 4, 5). The values of the smoothing factor were derived through results from an experimental pilot in which these values gave a large range of error rates centered on 3% error. This error threshold was chosen to be low because high tracking performance is desirable for model fitting, but the task should not be so easy that participants reach a performance ceiling. Each run completed by each participant used a new pseudorandom time-series generated at the difficulty level specified by the experimenter.

The position control model used in this program is adapted from PCT (Powers, 1973; Powers, Clark, & McFardland, 1960; Powers, Clark, & Mcfarland, 1960). PCT is a biologically plausible theory of behavior, with roots in control systems theory. It states that organisms control their perceptions at referent goal states by varying their motor behavior. This is implemented by a negative-feedback architecture comprising the organism, the environmental variable that it desires to control (the controlled variable), and the feedback path (Marken, 2014). These are encapsulated in the four functions of the control architecture: the input function, the comparator function, the output function, and the environment (feedback) function. These functions have associated parameters, such as delays and gains, which are pre-specified in PCT.

These parameters are the key to individual differences as parameter values are optimized as an individual learns. One parameter, the reference value, represents the desired state of the controlled variable, which is compared to the current perception of the controlled variable. These parameters, embedded in functions, determine the dynamic relationship between input and output, and due to feedback, the effect of this output on system input. Thus motor output is a purposeful effort to reduce any difference between the perceived current state of the controlled environmental variable (such as the distance between a held cup of tea and one’s mouth when drinking), and the desired perceptual state of that variable (the cup to one’s lips) (Powers, 1973). In pursuit tracking a participant senses the discrepancy between the cursor and the target, and compares this difference to a desired perceptual relationship (target-cursor alignment), acting to eliminate this error through varying joystick movements.

In the PCT model of the participant in the tracking task (Fig. 2a) the input function (*i*) senses the controlled variable (target-cursor distance) and translates this to a perceptual signal (*p;* the perceived difference between the target (*T)* and cursor (*C*); see Eq. 1 below:

1 $$ p=C-T $$

The comparator function (*co*) compares this perceptual signal (*p*) to the reference signal (*r*), the desired state of the controlled variable, and results in an error signal (*e*); see Eq. 2:

2 $$ e=r-p $$

The error signal (e) drives the output at the output function (*o*). This output, in the model, is the simulated joystick position, which determines the cursor position. Calculation of the new output *o(t)* of the control unit is determined in the program by the following formula, which contains a leaky integrator to counteract the accumulation of output over successive iterations (Eq. 3):

3 $$ o(t)=o\left(t-1\right)+\left({K}_o\times e\left(t-\tau \right)-{K}_d\times o\left(t-1\right)\right)\times \varDelta t $$

Where *Δt* is the time increment on each iteration (16.7ms), *o(t-1)* is the value of the output at the previous iteration, and *e(t – τ)* is the error with input delay (*τ)* samples. The model has four alterable parameters: the reference value (*r*), input delay (*τ*), output gain (*K*_*o*_), and damping constant (*K*_*d*_). The reference value specifies the desired distance between the cursor and the target that the model is aiming to achieve. It is a positive or negative integer, expressed in pixels. The input delay parameter is an estimate of the lag, in samples, of the cursor behind the target over the run. The output gain is a constant that proportionally amplifies the output, estimated from the velocity at which *e* is cancelled. The damping constant sets the leakage rate of the leaky integrator. It is therefore a constant that multiplies the previous output reduce its effect in the calculation of the current output, damping the response of the model. Whilst in the organic controller the input function (*i*), output function (*o*), and environment function (*f*) would also have associated gains, the model simplifies these by setting both input and environment function gains to the integer 1. Thus, the output gain represents the total loop gain for the system, and the equation for the environment function is:

4 $$ C(t)=o(t)/1 $$

A simulation of a PCT model with adaptable gains for all three functions can be found in Living Control Systems III: The fact of control (Powers, 2008).

Parameters are estimated via an optimization routine in which each parameter in turn is varied recursively to increase the goodness of fit between model-simulated cursor and actual cursor positions, assessed by a least-squares procedure repeated 20 times or until a minimum root-mean-square error (RMSE) change is achieved. Parameters are each fitted in this way in the order: output gain (*K*_*o*_), reference value (*r*), input delay (*τ*), then damping constant (*K*_*d*_). This order is replicated five times with the latest estimations for each parameter used as initial values for the next recursive loop. The order in which the parameters are fitted was arrived at empirically (Powers, 2008). Further details of the PCT model and TrackAnalyze program can be found in the appendices of Living Control Systems III: The Fact of Control (Powers, 2008).

#### Edinburgh handedness inventory

For completeness in characterizing the demographics of our sample, we collected data on the handedness of participants. For this the Edinburgh Handedness Inventory short form (Veale, 2014) was used. It is a four-item questionnaire in which participants indicate which hand they would usually use to complete everyday activities on a five-point Likert-type scale ranging from always left to always right. A global score indicates whether the individual is left-handed, mixed-handed or right-handed.

### Procedure

In the first session, participants read the instruction sheet explaining the pursuit-tracking task, and gave informed consent to take part. They completed the Edinburgh Handedness Inventory and performed two practice runs to familiarize themselves with the pursuit-tracking task. Participants completed a difficulty titration procedure, the purpose of which was to ensure that each participant was well practiced at the task, and to standardize the tracking error rate across the sample of participants. The latter was necessary because the accuracy in the fit of the simulated cursor movements to the actual cursor movements (model RMSE) was affected by the accuracy of the fit in actual cursor movements to the target pattern (tracking RMSE) for the run being modelled. Thus the variability in task performance was reduced by standardizing the error rate, which enabled greater comparability of model simulation accuracy between individuals. Participants completed sets of three runs over the five different target difficulty levels (determined by the smoothing factors). The highest difficulty level at which the participant produced a tracking RMSE error below 3% on all three runs was selected for the duration of that participant’s experiment. This procedure ensured that the task was equally difficult for each participant despite individual differences in participants’ performances. The threshold 3% scaled tracking RMSE was decided on as this was a typical error rate in pursuit tracking (Powers, 1978, 2008).

Following difficulty titration, participants started the 15 training runs, and after these, the 15 post-training runs. One week later, at the start of Session 2, participants received task instructions again and completed 15 follow-up runs. The design is summarized in Fig. 1. For each run in each of the three blocks a new pseudorandom target time-series was generated. Participants were thus administered different target time series from one another. No participant completed the same target time series more than once.

### Analyses

Prior to analysis, outliers were excluded from the dataset. This was necessary to control for tracking error as model fitting accuracy is extremely sensitive to participants’ initial tracking errors. *A priori*, we planned to exclude participants if the mean tracking error for each participant was higher than three standard deviations above the mean tracking error of the participant sample. All analyses were conducted using data analysis package IBM SPSS 22.

#### Analyses of intra-individual consistency and inter-individual differences

We conducted Cronbach’s alpha tests (Cronbach, 1951) for each parameter to determine whether participants’ parameter estimates were stable trial-to-trial, and over 1 week. Parameter estimates were generated for each run in all three blocks, totaling 45 estimates of each parameter for each participant. In addition, a mean measurement, absolute-agreement, two-way mixed effects model was used to calculate the intra-class correlation coefficient for each parameter. The analysis was replicated in the subgroup of 13 participants that completed the task at difficulty level 2.

We conducted a factorial ANOVA for each of the four parameters (*τ, K*_*o*_ *, K*_*d*_ *, r)* to determine whether the parameter estimates differed between participants, replicating a previous analysis that tested the individual differences in parameter estimates of a model in a sample of ten participants (Viviani et al., 1987). In our factorial design, participant was an independent group factor with 20 levels (as there were 20 participants). Block was a repeated measures factor with three levels; training, post-training and follow-up. To determine whether any inter-individual differences in parameters were due to participants tracking targets at different difficulty levels (due to the difficulty titration procedure) we conducted a subgroup analysis on the data from the participants that completed the task at the most common difficulty level (difficulty 2, n = 13).

#### Contribution of parameters to model accuracy

To investigate the nature of the relationship between the estimated parameter values and the accuracy of that model in simulating the participant’s movements (across runs, blocks, and individuals), we conducted a polynomial regression analysis with each of the model parameters as the predictor variables and model RMSE as the outcome variable. This stepwise analysis aimed to reveal whether the relationship followed a linear, quadratic, or cubic pattern. The most appropriate model would be indicated by whether the R^2^ change significantly improved as the polynomial order increased.

Following selection of an appropriate regression model order (quadratic), a second stepwise regression was conducted to determine the contribution of each parameter to the quadratic model. Parameters were added in a stepwise fashion; parameters were added in descending order of occurrence in tracking control models: output gain, input delay, damping constant, and finally reference value. We opted to add the reference value last because we intended to test whether this parameter is essential to accurate model performance. In PCT (and contrasting with other theories) this parameter is set from within the system and can take non-zero values (Mansell & Marken, 2015). Adding this parameter to the regression model last would determine whether it contributed significantly to model accuracy after all other parameters had been added, and therefore whether this parameter improved the control model.

It was thought that as participants completed the task at different difficulty levels this might confound the regressions as the different task constraints may influence parameter optima and show different distributions. Consequently we repeated the above analyses within the subgroup.

#### Accuracy of individual computational models

An individual model was developed for each participant by taking the mean of the estimates for each parameter across the 15 runs of the training block. To test whether these individual models accurately simulated the participant’s tracking movements at post-training and follow-up (validation), we compared each participant’s cursor positions as simulated by the model during the post-training and follow-up runs to the same participant’s actual cursor positions during these blocks; the model RMSE.

#### Individual specificity of the computational models

We aimed to test the hypothesis that a participant’s tracking data would be more accurately simulated by the participant’s individual model than by other participants’ models. For simplicity, the analysis procedure is outlined for the tracking data from participant 1 (P1). This procedure would be repeated for each of the 20 participants.

The model generated from P1’s training data was fit to P1’s 15 tracking runs in the post-training and follow-up blocks. Averaging the accuracy across the 15 runs of each block yielded the P1 “self” model RMSE value for each block. To generate the “other” model RMSE, the other 19 models (derived from P2-P20 training data) simulated each of the 15 tracking runs in P1’s post-training, and follow-up blocks. This yielded 19 model RMSE means and standard deviations of the error around these means in each block (one for each model fit to the 15 P1 runs). A weighted mean of these 19 model means was calculated, resulting in a single aggregate “other” model RMSE for each block.

The mean RMSE fit for each of the 19 “other” models to P1’s data was weighted according to the reciprocal of its associated standard deviation. Thus larger standard deviations in model fits were assigned smaller weights. This measure was taken to control for large standard deviations in simulation error relative to the mean error rate across the 15 runs. The weighted averages were calculated with the following Eq. 5:

5 $$ {x}_{weighted}=\frac{\sum_{i=1}^n\left({x}_i\times {w}_i\right)}{\sum_{i=1}^n{w}_i} $$

Where *x*_*weighted*_ is the aggregate RMSE to each individual’s tracking runs in each block, *x* is the mean RMSE when each model simulated the 15 tracking runs in that block, and *w* is the weight allocated to each mean as a function of the standard deviation around the mean RMSE within each block. This procedure was repeated for each participant’s tracking data such that it yielded 20 “self” model fits and 20 aggregate “other” model fits.

To compare the simulation accuracy of self and other models, we conducted a 2×2 repeated measures ANOVA. The first repeated factor was model type: self versus other. The second repeated measures factor was block and had two levels: post-training and follow-up.

The same analysis was conducted with the sample subgroup that included only participants tested on the most common difficulty level (difficulty 2, 13 participants) to determine whether any differences between “self” and “other” model fits were an artefact of the participants tracking at different difficulty levels.

## Results

Complete data were collected from 20 participants. Five participants were male and 15 were female. Sixteen participants were right-handed, four were mixed-handed. Mean age was 23.8 years (SD = 6.59 years).

There were no outliers among participant data; all participants’ data were included in the analysis. Tracking and model RMSE were positively skewed. Following a log transformation a normal distribution was observed in participant tracking and model RMSE. The number of participants that completed the experiment at each difficulty level was as follows: Difficulty 1, four participants; Difficulty 2, 13 participants; Difficulty 3, three participants.

### Analyses of intra-individual consistency and inter-individual differences

Cronbach’s alpha coefficients for consistency in estimated parameter values were 0.921 (subgroup: 0.858) for input delay, 0.976 (subgroup 0.886) for output gain, 0.880 (subgroup 0.852) for damping constant, and 0.920 (subgroup 0.810) for reference value, indicating that all parameter estimates were highly consistent within individuals over the course of the experiment. Results of the intra-class correlations can be found in Table 1. Examination of lower bounds of the confidence interval for each parameter, following the interpretation guidelines of McGraw and Wong (McGraw & Wong, 1996), indicated output gain showed good reliability, whilst input delay, damping constant and reference value showed moderate reliability. The same pattern of results was observed in the subgroup intra-class correlation analysis.

**Table 1:** Intra-class correlation coefficients for each of the parameter values using average-rating, absolute-agreement, and two-way mixed-effects model

Average measures	Intraclass correlation	95% confidence interval		F test with true value 0			
		Lower bound	Upper bound	*F*	Df1	Df2	*P*
Input delay	.881	.751	.949	8.34	19	38	<.001
Output gain	.914	.820	.963	11.75	19	38	<.001
Damping constant	.866	.717	.943	8.34	19	38	<.001
Reference value	.824	.628	.925	5.50	19	38	<.001

**Fig. 3:**
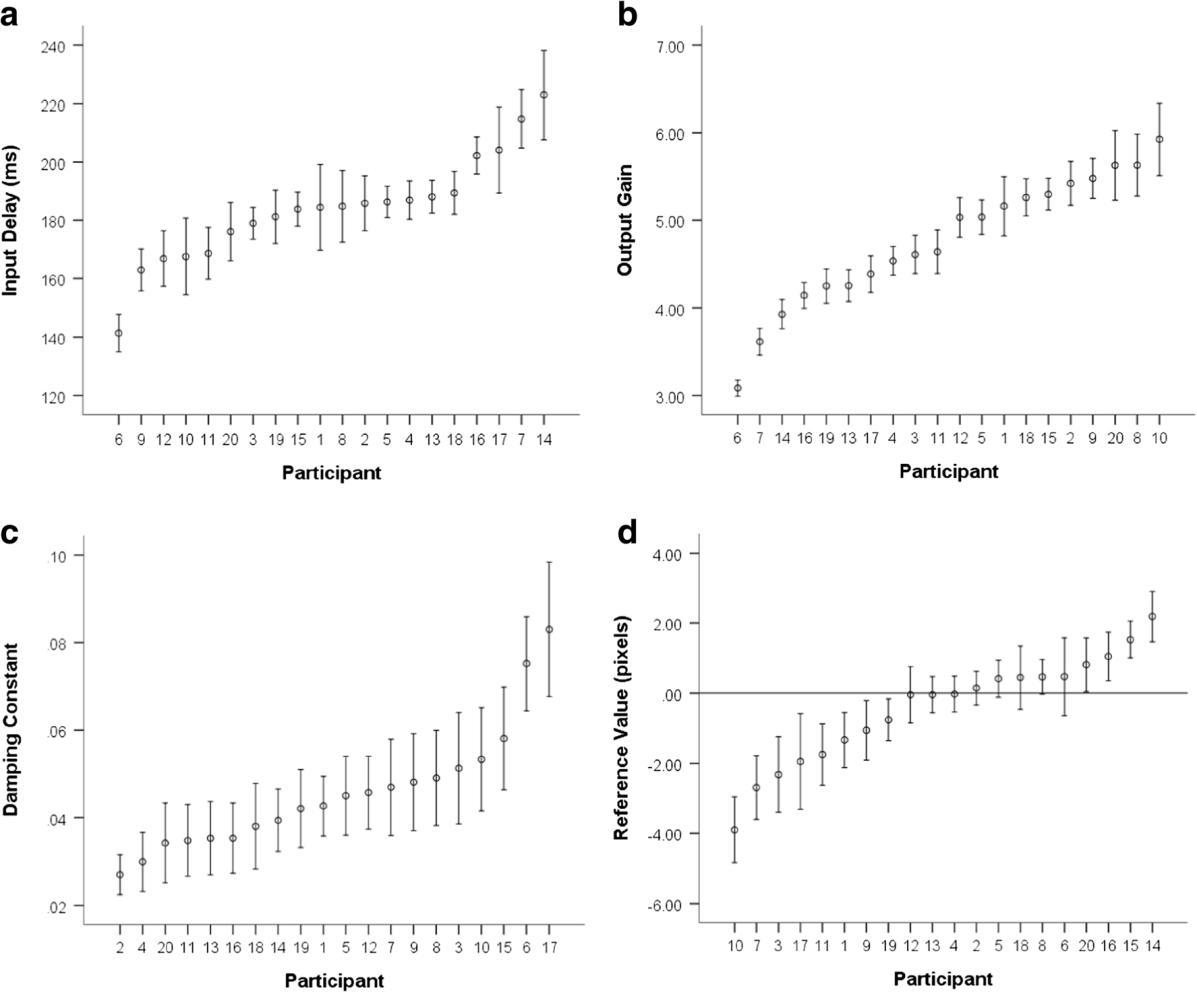
Error bar plots showing the mean value and standard deviations of parameter estimates across all trials for each participant. **a** Input delay (*τ*), **b** Output gain (*K*_*o*_), **c** Damping constant (*K*_*d*_), and **d** Reference value (*r*)

The ANOVAs indicated significant differences in all parameters between participants across blocks. In the sub-analysis this was also found to be the case. Interactions between the factors of participant and block were also significant (Table 2). Post-hoc one-way ANOVAs within each block revealed individual differences in parameter estimates between participants within each block for all parameters. Inspection of the effect sizes in Table 2 revealed that the largest individual differences between participants were in estimates of output gain (*K*_*o*_). Figure 3 shows four graphs, each showing the mean and 95% confidence interval of parameter estimates of the input delay (*τ*) and output gain (*K*_*o*_), damping constant (*K*_*d*_), and reference value (*r*) for each participant. Both inter-individual variability and intra-individual consistency can be observed. This pattern is pronounced in the output gain condition where both intra-individual consistency and inter-individual variability are highest.

**Table 2:** Results of the 2×3 factorial analyses and associated post-hoc ANOVAs for each parameter

Factor	*df1*	*df2*	*F*	*p*	Partial η^2^
Input delay (*τ*)					
Participant	19	277	12.62	< .001*	.464
Block	2	554	1.29	.277	.005
Interaction	38	554	1.98	< .001*	.120
Post-hoc: training					
Participant	19	299	8.77	<.001*	
Post-hoc: post-training					
Participant	19	298	3.46	<.001*	
Post-hoc: follow-up					
Participant	19	297	7.75	<.001*	
Output gain (*K*_*o*_)					
Participant	19	277	33.60	< .001*	.697
Block	2	554	5.63	.004*	.020
Interaction	38	554	4.18	<.001*	.223
Post-hoc: training					
Participant	19	299	18.47	<.001*	
Post-hoc: post-training					
Participant	19	298	11.75	<.001*	
Post-hoc: follow-up					
Participant	19	297	20.15	<.001*	
Damping constant (*K*_*d*_)					
Participant	19	277	21.39	< .001*	.595
Block	2	554	6.26	.002*	.022
Interaction	38	554	1.48	.036*	.092
Post-hoc: training					
Participant	19	299	5.30	<.001*	
Post-hoc: post-training					
Participant	19	298	5.86	<.001*	
Post-hoc: follow-up					
Participant	19	297	7.90	<.001*	
Reference value (*r*)					
Participant	19	277	15.71	< .001*	.519
Block	2	554	0.44	.645	.002
Interaction	38	554	2.85	<.001*	.163
Post-hoc: training					
Participant	19	299	8.99	<.001*	
Post-hoc: post-training					
Participant	19	298	6.06	<.001*	
Post-hoc: follow-up					
Participant	19	297	6.61	<.001*

### Contributions of parameters to model accuracy

Stepwise regressions were conducted to determine whether a linear, quadratic, or cubic model best fit the available data. The results of the model fit can be found in Table 3. Examination of the R^2^ change values associated with the models revealed that the quadratic equation accounted for a significantly more of the variance in accuracy than did the linear model. The cubic equation also made a significant contribution to the regression fit. However the R^2^ change was very small and the F value was reduced relative to the quadratic model. Inspection of the data plots (not reproduced in this article) showed that the third-order curves did not deviate from the general path of the second-order curves.We therefore opted to use a quadratic model for the stepwise regression to determine parameter contributions.

**Table 3:** Comparison of polynomial regression models where parameters predict model accuracy

Model	*F*	*p*	R	R^2^	Change statistics	
					R^2^ change	*p*
1 Linear	44.18	0.000	0.407	0.165	-	-
2 Quadratic	75.11	0.000	0.635	0.404	0.238	0.000
3 Cubic	51.75	0.000	0.642	0.413	0.009	0.009

Model Predictors: Output gain, Input delay, Damping constant, Reference value (all models)

Investigation of the contribution of each parameter to the quadratic model revealed that the addition of each parameter as a predictor of model performance increased the R^2^ fit significantly (Table 4). This was also found when we conducted the analysis as a cubic regression. Tests of multicollinearity revealed that parameters fell within the acceptable range, with guidelines stating a variance inflation factor (VIF) threshold of 5–10 as a cut-off (Craney & Surles, 2002). VIF: Output gain, 1.081; Input delay, 1.086; Damping constant, 1.087; Reference value 1.099.

**Table 4:** Stepwise regression to determine parameter contribution to model accuracy

Model predictors	*F*	*p*	R	R^2^	Change Statistics	
					R^2^ change	*p*
Output gain	23.91	<.001	.273	.074	-	-
Output gain, input delay	34.87	<.001	.436	.190	.116	<.001
Output gain, input delay, damping constant	56.53	<.001	.604	.365	.174	<.001
Output gain, input delay, damping constant, reference value	51.75	<.001	.642	.413	.048	<.001

Both analyses were repeated in the subgroup of 13 individuals that completed the experiment at difficulty level 2. This yielded the same pattern of findings: significant R^2^ change for cubic over quadratic over linear models, and parameters contributed significantly to regression model accuracy for both quadratic and cubic models.

### Accuracy of individual computational models

The average simulation error (model RMSE) when the 20 models generated from data at training simulated the cursor movements of the participant from which they were derived (“self”) at post-training was 2.05% (SD = 0.37), 95% CI (1.88–2.22) and 1.82% (SD = 0.38), 95% CI (1.64–1.99) at follow-up. These values were in the same range as the error rate as that when models simulated the tracking runs on which the models were trained; the mean model RMSE when training models simulated training data was 1.85% (SD = 0.48%).

### Individual specificity of the computational models

We hypothesized that models would be individual-specific, that is, a model of a participant’s performance would simulate that participant’s tracking movements more accurately than models generated from other participant’s tracking. The mean model RMSE of aggregate “other” models to participants actual tracking data at post-training was 2.11% (SD = 0.35), 95% CI (1.94–2.27) and 1.91% (SD = 0.42), 95% CI (1.71–2.11) at follow-up.

The 2×2 repeated measures ANOVA revealed that the main effect of model was significant *F*_(1, 19)_ = 5.76, *p* = .027, partial η2 = .232; “self” model fits were more accurate than the aggregate “other” model fits. The main effect of block was also significant *F*_(1, 19)_ = 8.45, *p* = .009, partial η^2^ = .308. Models generated during training more accurately fit the follow-up data than the post-training data. There was no interaction between model and block.

Within the subgroup who completed a task of identical difficulty, the mean simulation accuracy when 13 models fit to self tracking data was 1.92% (SD = 0.29), 95% CI (1.76–2.10) at post-training and 1.71% (SD = 0.25), 95% CI (1.55–1.87) at follow-up. The mean accuracy when the aggregate other models fit tracking data was 2.01% (SD = 0.30), 95% CI (1.82–2.19) at post-training and 1.77% (SD = 0.23), 95% CI (1.62–1.91) at follow-up. The subgroup 2× repeated measures ANOVA (13 participants) resulted in the same pattern of findings; firstly, a significant main effect of model *F*_(1,12)_ = 25.59, *p* < .001, partial η2 = .681, self models showed reduced error relative to other models. The effect of block was also significant *F*_(1,12)_ = 11.19, *p* = .006, partial η2 = .483. Models more accurately fit follow-up than post-training data. There was no interaction between model and block.

## Discussion

We found that when model parameters were estimated directly from participant pursuit tracking of pseudorandom targets, these estimated parameter values were consistent over time within individuals, but varied between individuals. These parameters accounted for a large proportion of the variance in model simulation accuracy and shared a curvilinear relationship. Moreover, when models generated from a participant’s pursuit tracking data at one time point simulated their performance at a later time point (model validation), these simulations were highly accurate, even after 1 week. Finally we demonstrated that a model produced from an individual’s performance more accurately simulated the cursor movements of that participant than did other individuals’ models.

### Analyses of intra-individual consistency and inter-individual differences

The results support our hypothesis that parameter estimates would be consistent over time within participants. This was demonstrated by the high internal consistency in parameter estimates over the training, post-training, and follow-up blocks within participants according to Cronbach’s alpha coefficients, and moderate to high intra-class correlation coefficients. This indicated that control parameters remained stable over 1 week for each participant. Factorial analyses conducted in this study found individual differences in parameter estimates. These findings support similar findings of individual differences in estimated model parameters between individuals (Viviani et al., 1987) that constitute robust idiosyncratic pursuit tracking strategies (Franks et al., 1982; Miyake et al., 2001). The current study demonstrated that such differences persisted even when data were collected over 1 week, which is evidence that control parameters remain stable over time within an individual. A model architecture was successfully parameterized to characterize individuals’ strategies in the pursuit-tracking task, even though the movements required to track the pseudorandom target varied between trials. Interestingly, output gain was the most variable parameter between individuals, and had the highest consistency within individuals. Consequently, it had the most discriminatory power and was the strongest indicator of an individual’s control strategy. It is unclear from this experiment alone whether this is a task-specific parameter or alternatively whether a higher estimated output gain in tracking tasks would be associated with a higher output gain in other task paradigms.

### Contribution of parameters to model accuracy

We hypothesized that estimated parameters for each run would share a quadratic relationship with the model simulation error when those parameters were used to fit the tracking data. Investigation of the nature of the relationship revealed that the parameters did share a curvilinear relationship with model simulation error. Both the addition of quadratic (second-order), and cubic (third-order) regression parameters improved the fit to the data significantly. The second-order regression model resulted in a large improvement over the fit of the linear regression model and a third-order regression model only negligibly (though significantly) increased in fit to the data over the second-order regression model. The presence of such curvilinear peaks in simulation accuracy for each parameter indicates that there may be an optimal control strategy in the task on which skilled trackers are converging. Therefore these optima in parameter space are identified by the reorganization algorithm when control models are optimized to the tracking data.

When each parameter was added in a stepwise fashion to determine their individual contribution to performance, each addition improved the fit of the model to tracking performance. The addition of damping constant provided the largest improvement to model fit. The addition of the reference value parameter to the regression model (after all other parameters had been entered) yielded a significant increase in the proportion of variance in simulation accuracy explained by the regression model. This indicates that the unique PCT reference parameter made an individual contribution to explaining the variance in performance. The reference value might be assumed to be zero, as the task requires participants to minimize position error between the target and cursor. Yet it remained a key parameter in the control model, and demonstrates that referent perceptual goals are fundamental in motor performance and should be included within control models.

### Accuracy of individual computational models

Model simulation error was very low at both post-training and follow-up. In fact, simulation error was lower when models simulated follow-up data than post-training data, despite the temporal proximity of training and post-training data collection. Participant tracking error was also increased at post-training relative to follow-up. We suggest that this increase in error might be due to participant fatigue, as they had to complete more than 30 1-min runs in succession, and that this increased tracking error reduced the model simulation accuracy as a consequence. One might reasonably expect that the model would more accurately simulate the tracking data on which it was trained than the new targets in the post-training and follow-up blocks. However, in this study the simulation error rate across training, post-training, and follow-up were virtually the same. This would appear to provide strong evidence that the parameters are trait-like features of the individual independent of target movements. This supports the hypothesis that models generated from an individual’s performance during training highly accurately simulated their later tracking movements. These findings are consistent with previous reports that dynamic models accurately simulate human control movements in pursuit-tracking tasks (Abdel-Malek & Marmarelis, 1988; Powers, 1978, 1989; Viviani et al., 1987), even when models are validated with data from a later testing session (Bourbon, 1996; Bourbon et al., 1990).

### Tests of individual specificity of the models

To establish whether models were individual-specific, we tested each model’s simulation accuracy to participants own data and the data of other individuals. We found that although the difference was small in magnitude, participants’ own models consistently simulated their own data more accurately than did other participants’ models. This difference was maintained and in fact increased when the analysis was repeated in the subgroup of participants who completed the task at the same difficulty level, indicating that the difference in simulation accuracy by self and other models was not as a result of participants tracking under different task constraints. Thus model parameters optimized from participants’ data at one time point can be used to simulate that individual’s performance 1 week later, with higher accuracy than a model not optimized to that individual. This is a remarkable finding when considering the robustness of the model to differences in human tracking and is, to our knowledge, the first formal test of individual model specificity.

Whilst previous studies have highlighted the individual differences between control strategies utilized by individuals in tracking tasks, the current study demonstrates that models optimized to individual tracking data can characterize these idiosyncratic strategies that persist over time in individuals practiced in such tasks. Tests of replicability within an individual should be a benchmark validity criterion when evaluating models of human behavior (Smith & Conrey, 2007), as it is in other fields of psychology in which trait-level constructs are measured. In such cases, it is recognized that in order for hypothesized task- and individual-specific factors to be valid, they must demonstrate test-retest reliability (Chaplin, John, & Goldberg, 1988; Oppenheim & Oppenheim, 1992).

Individual models of pursuit tracking performance may be effective tools in the assessment and treatment of motor deficits following neurologic injury. The pioneering research in analysis of estimated model parameters for people with Parkinson’s (Aiman Abdel-Malek et al., 1988; Au et al., 2010; Oishi et al., 2010, 2011) indicates that models might be used to assess bradykinesia and other deficits in this group (Allen et al., 2007). In therapeutic settings, upper-limb assistive robotic devices provide force assistance in upper limb movements to those with neurological motor impairments, often during virtual-reality pursuit-tracking tasks (Maciejasz, Eschweiler, Gerlach-Hahn, Jansen-Troy, & Leonhardt, 2014). Whilst assistive robotics often collect kinematic data which may help to assess symptom severity, individual models may be critical for delivering idiosyncratic rehabilitation regimes to people with neurological conditions. These individuals exhibit heterogeneous symptoms and outcomes (Kwakkel, Kollen, & Lindeman, 2004; Reinkensmeyer, Emken, & Cramer, 2004) and may use different motor strategies at different points in the recovery process (Fitts, 1964). Individual models may be useful to identify and treat specific deficits through tailored assistance or resistance control regimes (Marchal-Crespo & Reinkensmeyer, 2009).

### Strengths and limitations

This first formal test of individual-specificity over time has found that position control models could provide idiographic simulations of human behavior in pursuit-tracking tasks. However, the magnitude of the difference between idiographic and general models was small. The most likely explanation for this is that the parameter estimates are affected by the task constraints, and so participants converged on an optimum strategy in this task. This is supported by the finding that different target motion patterns and different target frequencies elicit characteristic tracking profiles and estimated model parameters in participants (Abdel-Malek & Marmarelis, 1988; Poulton, 1952b; P Viviani & Mounoud, 1990). This highlights an important caveat. Whilst we were able to detect the consistencies in control parameters and tracking strategies over time in this experiment, this was enabled by experimental control of the task constraints and demands, apparatus, and goal. If task demands were to vary suddenly in the experiment this would likely introduce variability in the control parameter estimates and performance. With repeated practice on the new task, performance and parameters would become more consistent as the individual learns a new control strategy (Franks et al., 1982). The model, as a model of a well-practiced participant, would require further optimization to perform well within the new task constraints. Exactly how much the task could be changed before the model fails to accurately simulate performance is uncertain.

We used a limited range of low target velocities in this experiment, and this resulted in participants achieving a near-ceiling performance. Higher velocity target movements would be necessary to comprehensively test individuals’ transient dynamics (Abdel-Malek & Marmarelis, 1990), which might expose further individual differences. The frequency content of targets could be controlled in future experiments by summation of sinusoids of different frequencies to ensure a sufficiently wide bandwidth within each pursuit run (Roth, Zhuang, Stamper, Fortune, & Cowan, 2011). In the current study, we manipulated the rate of change of the target to be tracked between different participants. This was in order to ensure that the difference between model simulation performances was not a result of their different levels of ability, and therefore tracking accuracy. However, this introduced a potential confound; participants completing the task under different task constraints (difficulty levels) could account for differences in parameter estimates, as has been reported in previous studies (Neilson et al., 1993; Notterman & Tufano, 1980; Viviani & Mounoud, 1990). We therefore repeated our analyses with a subgroup of 13 participants who completed tracking runs at a similar accuracy level to one another *and* under the same task constraints (Difficulty level 2). All main hypotheses were confirmed in the analyses of subgroup data, providing evidence that the pattern of results attained was not a consequence of either potential confound.

The model architecture used in this study was a simple first-order position control model with state delay, representing a single PCT control unit (Powers, 1973, 1978, 2008). This was chosen because it had been previously shown to accurately simulate perceptuo-motor behavior during a tracking task (Bourbon, 1996; Bourbon et al., 1990; Marken, 1991; Powers, 1978, 1989, 2008) and had a biologically feasible conceptual foundation (Powers, 1973). However, it is by no means a comprehensive model of human motor control. Rather it attempts to demonstrate that parameterization of such control architectures is useful to discriminate and simulate individual performance, regardless of whether they accurately specify how this would be achieved within human sensory and motor systems. It therefore follows that other model architectures may be more appropriate or accurate in simulating both neurologically atypical and healthy individuals. System identification can be used to find the best-fitting model, for example (Neilson et al., 1993; Oishi et al., 2010, 2011). In addition, there are known relationships between the non-independent parameters of the PCT control loop. For example, input delay and output gain are negatively correlated and therefore at high values of delay, a high output gain produces an oscillatory response. The damping constant and output gain parameters share a positive relationship; higher output gains require higher damping constants to avoid oscillatory behavior. Whilst beyond the focus of this article, these relationships have implications for model fitting, as different optimization routines (order and method, number of iterations) might affect the efficiency of the search of the parameter space, and consequently result in different parameter value combinations.

Critically, we investigated pursuit tracking in one-dimension, and the application to assistive robotics for neurorehabilitation would require extension to two- and three-dimensional tracking tasks and different target movement patterns (Engel & Soechting, 2000; Marken, 1991; Viviani et al., 1987; Viviani & Mounoud, 1990). Moreover, control of other perceptual variables may increase simulation accuracy, such as target-cursor angle (Marken, 2014) or target-cursor velocity difference (Johnson, Howe, & Chang, 2013; Proteau & Masson, 1997; Viviani et al., 1987; Viviani & Mounoud, 1990). Future studies should aim to elucidate individual control strategies under different task constraints, and their stability over time, particularly in populations with neurological conditions.

## Conclusions

In summary, we demonstrated that a negative-feedback computational model architecture can be optimized to characterize and accurately simulate an individual’s tracking data over time. Estimated control parameters were highly consistent over time, whilst individual differences in control strategies were discriminated by the computational model. All model parameters contributed to the accuracy of PCT models to fit human tracking data. Moreover, even when the target patterns differ from trial to trial, individual computational models very accurately simulate the movements of the individual from which they were derived. We argue that establishing the test-retest reliability in parameter estimates and simulation accuracy should be an essential criterion for computational models of human performance.
